# Endocrine Requirements for Oocyte Maturation Following hCG, GnRH Agonist, and Kisspeptin During IVF Treatment

**DOI:** 10.3389/fendo.2020.537205

**Published:** 2020-10-06

**Authors:** Ali Abbara, Tia Hunjan, Vu N. A. Ho, Sophie A. Clarke, Alexander N. Comninos, Chioma Izzi-Engbeaya, Tuong M. Ho, Geoffrey H. Trew, Artsiom Hramyka, Tom Kelsey, Rehan Salim, Peter Humaidan, Lan N. Vuong, Waljit S. Dhillo

**Affiliations:** ^1^Section of Endocrinology and Investigative Medicine, Imperial College London, Hammersmith Hospital, London, United Kingdom; ^2^IVFMD, My Duc Hospital, Ho Chi Minh City, Vietnam; ^3^In vitro Fertilization Unit, Hammersmith Hospital, Imperial College Healthcare NHS Trust, London, United Kingdom; ^4^School of Computer Science, University of St Andrews, St Andrews, United Kingdom; ^5^The Fertility Clinic, Skive Regional Hospital and Faculty of Health, Aarhus University, Aarhus, Denmark; ^6^University of Medicine and Pharmacy at Ho Chi Minh City, Ho Chi Minh City, Vietnam

**Keywords:** trigger, oocyte maturation, fertility, progesterone, *in vitro* fertilization treatment

## Abstract

**Objective:**

The maturation of oocytes to acquire competence for fertilization is critical to the success of *in vitro* fertilization (IVF) treatment. It requires LH-like exposure, provided by either human chorionic gonadotropin (hCG), or gonadotropin releasing hormone agonist (GnRHa). More recently, the hypothalamic stimulator, kisspeptin, was used to mature oocytes. Herein, we examine the relationship between the endocrine changes following these agents and oocyte maturation.

**Design:**

Retrospective cohort study.

**Methods:**

Prospectively collected hormonal data from 499 research IVF cycles triggered with either hCG, GnRHa, or kisspeptin were evaluated.

**Results:**

HCG-levels (121 iU/L) peaked at 24 h following hCG, whereas LH-levels peaked at ~4 h following GnRHa (140 iU/L), or kisspeptin (41 iU/L). HCG-levels were negatively associated with body-weight, whereas LH rises following GnRHa and kisspeptin were positively predicted by pre-trigger LH values. The odds of achieving the median mature oocyte yield for each trigger were increased by hCG/LH level. Progesterone rise during oocyte maturation occurred precipitously following each trigger and strongly predicted the number of mature oocytes retrieved. Progesterone rise was positively associated with the hCG-level following hCG trigger, but negatively with LH rise following all three triggers. The rise in progesterone per mature oocyte at 12 h was greater following GnRHa than following hCG or kisspeptin triggers.

**Conclusion:**

The endocrine response during oocyte maturation significantly differed by each trigger. Counter-intuitively, progesterone rise during oocyte maturation was negatively associated with LH rise, even when accounting for the number of mature oocytes retrieved. These data expand our understanding of the endocrine changes during oocyte maturation and inform the design of future precision-triggering protocols.

## Introduction

The World Health Organization recognizes subfertility as the fifth most serious global disability, affecting 1 in 6 couples (1). *In vitro* fertilization (IVF) is a supraphysiological process that simulates many of the physiological processes apparent during the natural menstrual cycle (2). The number of IVF cycles carried out annually is increasing (3), however 11.8% of cycles commenced did not progress to oocyte retrieval (3). Indeed, a recent international priority setting partnership designated the variation in oocyte number following IVF treatment as one of the top ten unresolved research uncertainties (4).

The “trigger” of oocyte maturation replicates the function of the mid-cycle ovulatory luteinizing hormone (LH) surge of the natural cycle (2) and provides LH-like exposure such that oocytes resume meiosis and advance to the metaphase II stage of development to acquire competence for fertilization (5). This LH-like activity can be provided by human chorionic gonadotropin (hCG), gonadotropin releasing hormone agonist (GnRHa), or kisspeptin. These agents have distinct mechanisms of action; namely hCG acts directly at ovarian LH receptors, GnRHa stimulates gonadotropin release from the pituitary gland, and kisspeptin stimulates the hypothalamus to induce release of endogenous GnRH (2). The hormone used to provide this LH-like exposure plays a determinant role in key outcomes affecting the success and safety of IVF treatment, including the ability to retrieve mature oocytes, luteal phase characteristics (impacting on pregnancy rates) and the occurrence of “ovarian hyperstimulation syndrome” (OHSS) (2).

HCG is the most widely used trigger in current practice, being applied in more than three quarters of cycles (6). As it shares the same alpha subunit, and has 85% homology of the beta subunit as native LH, hCG activates the LH receptor (7). HCG has a greater affinity for the LH receptor than native LH and activates distinct intracellular signaling, with a five-fold increased potency for cAMP activity in granulosa cells, whereas LH preferentially activates extracellular signal-related kinase 1/2 and protein kinase B (8). Overall, hCG has a greater steroidogenic action consistent with a critical role in supporting pregnancy, whereas LH has a stronger anti-apoptotic signal (2). Furthermore, hCG (t_1/2_ 28–29 h) has a longer half-life than native LH (t_1/2_ ~20 min), and thus also risks OHSS (2). GnRHa activates the pituitary gland to induce a shorter duration of LH exposure and thus has a lower risk of OHSS than hCG (9). Thus, GnRHa is usually reserved for patients at increased risk of OHSS, as more intensive luteal phase support is required to maintain pregnancy rates (9, 10).

More recently, kisspeptin has been used to safely induce oocyte maturation even in women at increased risk of OHSS (11–13). Kisspeptin stimulates hypothalamic GnRH neurons to release endogenous GnRH (14). Not all GnRH receptors on pituitary gonadotrophs are contiguous with GnRH neuronal terminals, and thus kisspeptin induces a tempered LH rise compared to equimolar doses of GnRH (15). Indeed, kisspeptin induced even fewer signs and symptoms of OHSS in women at increased risk of OHSS (16). This may in part be mediated through an additional direct ovarian action to reduce ovarian vascular endothelial growth factor (VEGF) production (13).

The ability to accurately quantify oocyte maturation facilitates the assessment of the minimum endocrine requirements for oocyte maturation. Different measures have been used to quantify oocyte maturation, including the absolute number of mature oocytes and the “oocyte maturation rate” (proportion of oocytes retrieved that are mature). However, suboptimal oocyte maturation can lead to fewer oocytes being retrievable, leading to a reduction in the denominator as well as the numerator, and thus the “oocyte maturation rate” is often preserved even in the context of suboptimal oocyte maturation. Consequently, we advocate the use of the “mature oocyte yield” defined by the number of mature oocytes expressed as a proportion of the number of follicles on the day of trigger most likely to yield a mature oocyte (17). While estimates for this follicle size have ranged from 10 to 14 mm (18–20), we have determined that follicles with a diameter of 12 to 19 mm on day of trigger are most likely to yield a mature oocyte (17). In the present study, we used this measure to evaluate the minimal endocrine requirements for LH-like activity (either LH or hCG level) for oocyte maturation following hCG, GnRHa and kisspeptin triggers.

## Materials and Methods

We conducted a comprehensive retrospective analysis of endocrine profiles prospectively collected from 499 IVF cycles triggered with either hCG, GnRHa, or kisspeptin. These cohorts were chosen as detailed endocrine data were collected following each trigger. The primary objective was to investigate the relationship between LH-like exposure after each trigger and the efficacy of oocyte maturation as quantified by the mature oocyte yield (MOY). The secondary objective was to assess determinants of the level of LH-like activity following each trigger and of the rise in progesterone during and after oocyte maturation.

### Study Participants

Participants were aged 18 to 42 years, body mass index (BMI) 18 to 30 kg/m^2^, and antral follicle count (AFC) 4 to 87. HCG and GnRHa-triggered cycles were oocyte donation research cycles conducted at My Duc Hospital in Vietnam, whereas kisspeptin-triggered cycles were conducted at Hammersmith Hospital in the UK. Data on kisspeptin have been published in (11–13). Data on GnRH agonist triggered cycles have been published in (21). Data on hCG/hormonal levels during the luteal phase have been published in (22, 23) and reviewed in (24).

### Study Approvals

All subjects gave written informed consent in accordance with the Declaration of Helsinki and Good Clinical Practice. Data from GnRHa triggered IVF cycles were obtained from a single-center randomized controlled trial conducted at My Duc Hospital, Ho Chi Minh City, Vietnam (21). The Institutional Review Board (IRB) reference number was NCKH/CGRH_01_2014 and ClinicalTrials.gov registration was NCT02208986. For the hCG case-series, the IRB reference number was NCKH/CGRH_09_2017, ethical approval reference number: 10/17/DD-BVMD and ClinicalTrials.gov Identifier: NCT03174691. For the kisspeptin trial, ethical approval was granted by the Hammersmith and Queen Charlotte’s Research Ethics Committee, London, UK (reference: 10/H0707/2), undertaken at the IVF Unit at Hammersmith Hospital under a license from the UK Human Fertilization and Embryology Authority (11–13) and registered on the National Institutes of Health Clinical Trials database (NCT01667406).

### Stimulation Protocol

Follicular stimulation was conducted using a GnRH antagonist co-treated cycle. FSH stimulation was in the form of 150 to 300 IU of follitropin-β for hCG-triggered, corifolliotropin alfa (Elonva; Merck Sharp & Dohme, UK) for GnRHa-triggered, and recombinant FSH (112.5–150 IU Gonal F, Merck Serono, Geneva, Switzerland) for kisspeptin-triggered cycles. The triggers recombinant hCG (250 µg) or GnRHa triptorelin (0.2–0.4 mg) were administered as soon as two follicles reached a size of ≥17 mm. The trigger kisspeptin-54 (6.4–12.8 nmol/kg as a single subcutaneous bolus or 19.2 nmol/kg as a split bolus over 10 h, Bachem Holding AG, Bubendorf, Switzerland) was administered once three follicles reached ≥18 mm. Patients who received two doses of kisspeptin were not included in analyses of parameters potentially affected by the second dose (outcomes after 10 h).

Hormonal parameters (LH, FSH, estradiol, and progesterone) were measured at regular intervals following hCG (0,12, 24, 36, 60, 84, 108, 132, 156, 180 h), GnRHa (0, 4, 12, 24, 36, 84, 132, 180, 228 h), and kisspeptin (0, 4, 10, 12, 14, 20, 36 h). HCG and GnRHa triggers were used in oocyte donation cycles allowing full examination of endocrine profiles during the luteal phase, whereas timepoints after 36 h following kisspeptin-triggered cycles were not examined due to contamination from luteal phase support. Only patients who had an ultrasound scan on the day of trigger were included in the analyses of the “mature oocyte yield.”

### Assays

GnRHa/hCG group: all samples were processed immediately and stored at −20°C. Serum hormone levels were determined using electrochemiluminescence immunoassay (ECLIA; Roche Cobas E 801, Roche Diagnostics, Germany). Lower level of quantification, inter-assay variability and intra-assay variability were LH 0.1 iU/L, 2–5% and 2–5%; hCG 0.1 iU/L, 2–5% and 2–5%; progesterone 0.5 ng/ml, 2–6% and 2–4%; and estradiol 5 pg/ml, 2–6%, and 2–4%, respectively. Kisspeptin group: serum LH, FSH, estradiol, and testosterone were measured using automated chemiluminescent immunoassays (Abbott Diagnostics, Maidenhead, UK). Interassay coefficients of variations were as follows: LH, 3.4%; FSH, 3.5%; estradiol, 3.4%; testosterone, 3.6%. Limits of detectability for each assay are as follows LH, 0.07 iU/L; FSH, 0.05 iU/L; estradiol, 70 pmol/L (19 pg/ml); testosterone, 0.08 nmol/L.

### Statistical Analysis

Normality was assessed by D’Agostino and Pearson test and equality of variances by F-test for two groups or the Brown-Forsythe test for multiple groups. Parametrically distributed continuous variables were analyzed by t-test for two groups, or one-way ANOVA with *post hoc* Tukey’s test for multiple groups, and non-parametrically distributed data by the Mann Whitney U test for two groups, or the Kruskal Wallis test with *post hoc* Dunn’s test for multiple groups. Simple linear regression was used to analyze the relationship between two continuous variables. Categorical variables were analyzed by logistic regression. Neural net and random forest models were used to quantify (a) the accuracy of the data when used to predict mature eggs and (b) the relative importance of LH/hCG as a predictor variable. Data were analyzed using GraphPad Prism version 8.0 and STATA version 14. *P*-value < 0.05 was regarded as indicating statistical significance.

## Results

Baseline characteristics are presented in **Supplementary Table 1**.

### Endocrine Profiles Following hCG, GnRHa, and Kisspeptin

**Figure 1:**
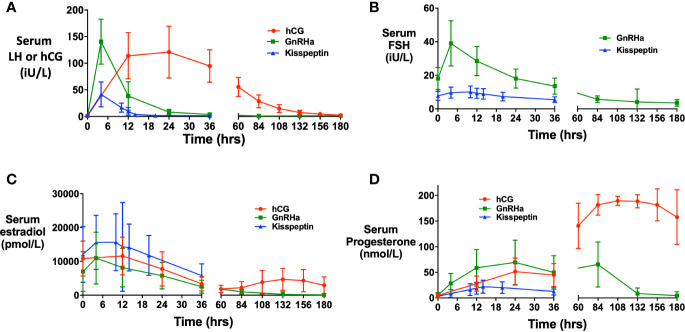
Hormonal responses after each trigger of oocyte maturation. **(A)** Mean (± SD) of change in serum human chorionic gonadotropin (hCG) levels (iU/L) over time in response to hCG (red), and change in serum LH after GnRH agonist (green) and kisspeptin (blue). **(B)** Mean (± SD) of change in serum FSH (iU/L) over time in after GnRHa (green) and kisspeptin (blue). **(C)** Mean (± SD) of change in serum estradiol (pmol/L) over time after hCG (red), GnRHa (green) and kisspeptin (blue). **(D)** Mean (± SD) of change in serum progesterone (nmol/L) over time after hCG (red), GnRHa (green) and kisspeptin (blue).

Mean LH levels peaked at ~4 h following GnRHa (140.4 iU/L) and kisspeptin (41.4 iU/L), whereas hCG levels (121.0 iU/L) peaked at ~24 h following hCG trigger (**Figure 1**). The initial progesterone rise (**Figure 1D**) peaked at 24 h following GnRHa (69.4 nmol/L) and hCG (51.4 nmol/L), and at ~14 h following kisspeptin (22.0 nmol/L).

### Serum LH/hCG Levels Following Trigger

**Figure 2:**
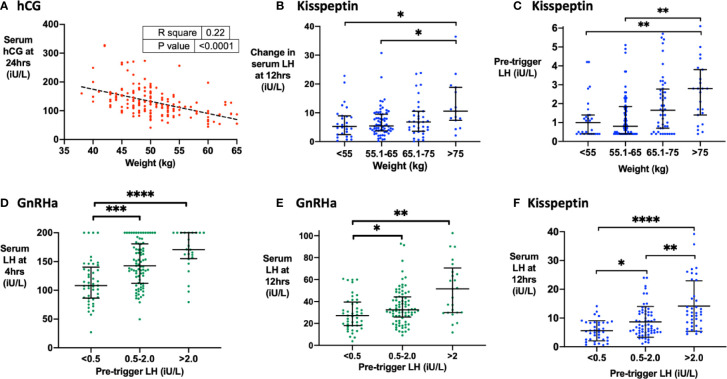
Factors that determine hCG levels after hCG trigger and the LH level after GnRHa or kisspeptin. **(A)** Pre-treatment weight (kg) negatively predicted serum hCG level at 24 h (iU/L) after hCG trigger by simple linear regression (n = 161). Serum hCG at 24 h (iU/L) = −4.21 X body weight (kg) + 343.1, r^2^ = 0.22, *P <* 0.0001. **(B)** Median (IQR) of change in serum LH at 12 h (iU/L) by categories of body weight (kg) after kisspeptin (n = 141). Categories were compared by the Kruskal-Wallis test with post-hoc Dunn’s multiple comparison test. **(C)** Median (IQR) of pre-trigger serum LH (iU/L) by categories of weight (kg) after kisspeptin (n = 173). Categories were compared by the Kruskal-Wallis test with post-hoc Dunn’s multiple comparison test. Three outliers were not shown on the graph. **(D)** Median (IQR) of serum LH at 4 h (iU/L) by categories of pre-trigger LH after GnRHa (n = 150). Categories were compared by the Kruskal-Wallis test with post-hoc Dunn’s multiple comparison test. **(E)** Median (IQR) of serum LH at 12 h (iU/L) by categories of pre-trigger LH after GnRHa is presented (n = 151). Categories were compared by the Kruskal-Wallis test with post-hoc Dunn’s multiple comparison test. Two outliers were not shown on the graph. **(F)** Median (IQR) of serum LH at 12 h (iU/L) by categories of pre-trigger LH after kisspeptin (n = 142). Categories were compared by Kruskal-Wallis test with post-hoc Dunn’s multiple comparison test. *P < 0.05, **P < 0.01, ***P < 0.001, ****P < 0.0001.

Body-weight negatively predicted hCG levels at 24 h following hCG trigger (*r* = −4.2, r^2^ = 0.22, *P* < 0.0001; **Figure 2A**). LH rise at 4 h (but not 12 h) was negatively predicted by BMI after GnRHa (r = −3.3, r^2^ = 0.05, P = 0.008), whereas LH rise at 12 h was greater in women with body-weight >75 kg following kisspeptin (**Figure 2B**). Kisspeptin was dosed using a weight-based regimen, which may in part explain this result. However, higher pre-trigger LH levels with increased body-weight could also contribute to the increased rise in LH in those with increased body weight (**Figure 2C**). Indeed, the pre-trigger LH level was the strongest predictor of LH rise following both GnRHa and kisspeptin. Specifically, LH rise at 4 h and 12 h following GnRHa (**Figures 2D, E**) and at 12 h following kisspeptin (**Figure 2F**) were positively predicted by pre-trigger LH level.

### Endogenous LH Levels Following hCG Trigger

HCG levels at 24 h were negatively associated with endogenous LH levels at 24 h (r = −5.46, r^2^ = 0.035, P = 0.018), but there was no significant association between hCG and LH levels at any later timepoint. Women with higher endogenous LH levels prior to hCG trigger continued to have higher LH levels after hCG-levels started to fall following hCG trigger (i.e. pre-trigger LH was positively associated with LH at 24 h following hCG; r 0.49, r^2^ 0.35, P < 0.0001).

### Effect of LH/hCG on Oocyte Maturation

Notably, there was little to no association between levels of LH-like activity and the number of mature oocytes after all three triggers. Indeed, neither hCG levels at 12 h, 24 h (**Supplementary Figure 1A**) or 36 h after hCG, nor LH rise at 4 h **(****Supplementary Figure 1B****)**, 12 h (**Supplementary Figure 1C**), or 24 h following GnRHa were associated with the number of mature oocytes retrieved. After kisspeptin, LH rise at 12 h (*P* = 0.048) (but not at 4 h or 36 h) was weakly associated with the number of mature oocytes retrieved (**Supplementary Figure 1D**).

**Figure 3:**
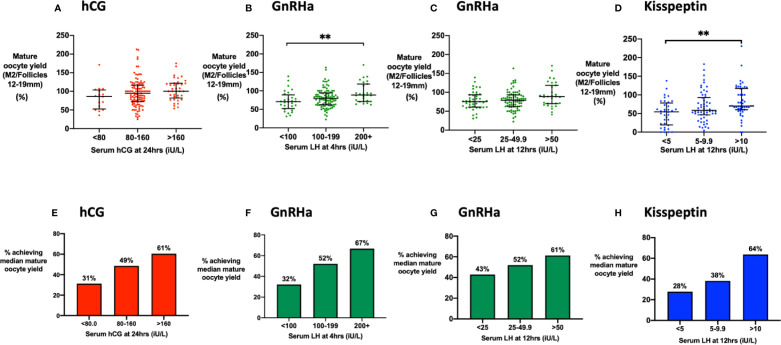
Mature oocyte yield by level of LH-like activity. **(A)** Median (IQR) of the mature oocyte yield (number of mature oocytes divided by the number of follicles of 12 to 19 mm in diameter on day of trigger) by categories of serum hCG at 24 h (n = 161). Categories were compared by the Kruskal-Wallis test with post-hoc Dunn’s multiple comparison test. **(B)** Median (IQR) of the mature oocyte yield by categories of serum LH at 4 h after GnRHa (n = 151). Categories were compared by Kruskal-Wallis test with post-hoc Dunn’s multiple comparison test. **(C)** Median (IQR) of the mature oocyte yield by categories of serum LH at 12 h after GnRHa is presented (n = 152). Categories were compared by Kruskal-Wallis test with post-hoc Dunn’s multiple comparison test. **(D)** Median (IQR) of the mature oocyte yield by categories of serum LH at 12 h after kisspeptin (n = 142). Categories were compared by the Kruskal-Wallis test with post-hoc Dunn’s multiple comparison test. **(E)** The proportion of women achieving the median mature oocyte yield with hCG used as a trigger were compared by categories of serum hCG at 24 h: <80 (n = 16), 80–160 (n = 107), > 160 (n = 38). Categories were compared by logistic regression: *P = 0.13*. **(F)** The proportion of women achieving the median mature oocyte yield with GnRHa were compared by serum LH at 4 h: <100 iU/L (n = 28), 100–199 iU/L (n = 96), >200 iU/L (n = 27). Categories were compared by logistic regression: The odds of achieving the median mature oocyte yield was increased by 4.22-fold (95% CI 1.37–13.02; P = 0.012) if LH at 4 h following GnRHa was at least 200 iU/L compared to <100 iU/L. **(G)** The percentage achieving the median mature oocyte yield with GnRH used as a trigger were compared by serum LH at 12 h: <25 iU/L (n = 42), 25–49.9 iU/L (n = 79), >50 iU/L (n = 31). Categories were compared by logistic regression *P* = 0.29. **(H)** The percentage achieving the median mature oocyte yield after kisspeptin by serum LH at 12 h: <5 iU/L (n = 18), 5–9.9 iU/L (n = 34), >10 iU/L (n= 58) was compared by logistic regression. The odds of achieving the median mature oocyte yield was increased by 4.6-fold (95% CI 1.4–14.6; *P* = 0.010) if LH at 12 h following kisspeptin was >10 iU/L compared to <5 iU/L. **P < 0.01.

We analyzed the cumulative “mature oocyte yield” (MOY; number of mature oocytes divided by the number of follicles of 12–19 mm on the day of trigger) to identify the threshold of hCG/LH level, beyond which there was unlikely to be any significant additional benefit to oocyte maturation from higher levels. Binary thresholds were ~80 iU/L for hCG at 24 h, ~25 iU/L at 12 h following GnRHa and ~10 iU/L at 12 h following kisspeptin (**Supplementary Figures 1E–H**). MOY was not significantly associated with the peak hCG level at 24 h (**Figure 3A**). LH at 4 h (**Figure 3B**) following GnRHa was associated with the mature oocyte yield (MOY), but not at 12 h (**Figure 3C**), or later timepoints. LH > 10 iU/L at 12 h following kisspeptin (but not at 4 h or 36 h) was associated with increased MOY (**Figure 3D**).

We investigated the reliability of triggering oocyte maturation at different hCG/LH thresholds by assessing the proportion of patients achieving the median MOY for each trigger. The odds of achieving the median MOY for hCG was increased by 3.4-fold (95% CI 0.97–11.7) in those with an hCG level >160 iU/L vs <80 iU/L (**Figure 3E**). For GnRHa, the odds of achieving the median MOY was increased by 4.2-fold (95% CI 1.4–13.0) if 4 h LH >199 iU/L vs <100 iU/L (**Figure 3F**). The proportion achieving the median MOY following GnRHa was 43% if LH at 12 h was <25 iU/L, 52% if 25 to 49.9 iU/L, and 61% if >50 iU/L (*P* = 0.29) (**Figure 3G**). For kisspeptin, the odds of achieving the median MOY was increased by 4.6-fold (95% CI, 1.4–14.6) if 12 h LH >10 iU/L vs <5 iU/L (**Figure 3H**).

Random forest models outperformed neural network models when predicting the number of mature oocytes from baseline characteristics, hormone levels and number of 12 to 19 mm follicles (88% accuracy of prediction to within 3 mature eggs for random forests; 57% accuracy for neural networks). Random forest accuracy fell from 88% to 83% when data on LH/hCG levels were not included.

We evaluated the area under the curve over 36 h for hCG levels following hCG trigger, or for LH following GnRHa or kisspeptin triggers, however this measure was not associated with either the number of mature oocytes, nor the MOY *(P>0.14* for all*)*. There was no significant difference in “oocyte maturation rate” or “fertilization rate” by category of hCG/LH following any trigger (**Supplementary Figure 2**).

### Progesterone Rise During Oocyte Maturation

Progesterone peaked at 24 h to 51.2 nmol/L following hCG trigger consistent with granulosa cell luteinization/oocyte maturation, before a subsequent greater rise at 108 h (4.5 days post-hCG) to 190.8 nmol/L corresponding to secretion from corpora lutea. Similarly, progesterone peaked to 69.4 nmol/L at 24 h following GnRHa, before a secondary rise to 66.4 nmol/L at 84 h (3.5 days) post GnRHa. Following kisspeptin, progesterone peaked to 22.3 nmol/L at 14 h (luteal progesterone levels were not assessed due to contamination from luteal phase support).

**Figure 4:**
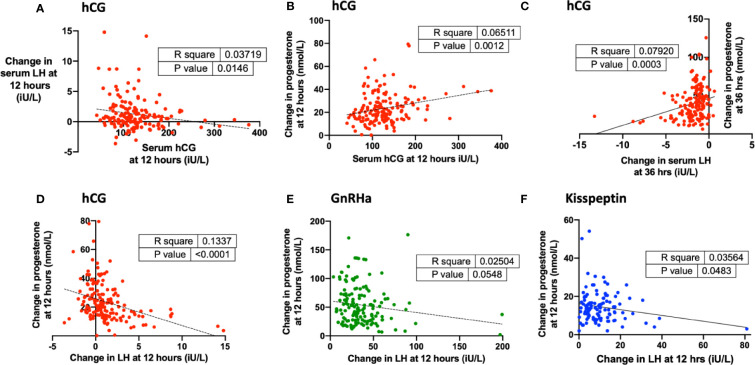
Endogenous LH levels after each trigger and relationship with progesterone rise during oocyte maturation. **(A)** Change in serum hCG at 12 h (iU/L) was negatively associated with change in serum LH at 12 h (iU/L) after hCG by simple linear regression (n = 161). Change in serum hCG at 24 h = −0.010 × change in serum hCG at 12 h + 2.47, r^2^ = 0.04, *P* = 0.01. **(B)** Serum hCG at 12 h (iU/L) was negatively associated with change in progesterone at 12 h (nmol/L) after hCG by simple linear regression (n = 159). Change in progesterone at 12 h (nmol/L) = 0.065 x serum hCG at 12 h (iU/L) + 15.3, r^2^ = 0.065, *P* = 0.001. **(C)** Change in serum LH at 36 h (iU/L) negatively predicted change in progesterone at 36 h (nmol/L) after hCG by simple linear regression (n = 160). Change in progesterone at 36 h (nmol/L) = 3.52 X change in serum LH at 36 h + 46.4, r^2^ = 0.08, *P* = 0.0003. **(D)** Change in LH at 12 h (iU/L) was negatively associated with change in progesterone at 12 h (nmol/L) after hCG by simple linear regression (n = 160). Change in progesterone at 12 h (nmol/L) = −1.86 × change in serum progesterone at 12 h + 25.85, r^2^ = 0.133, *P* < 0.0001. **(E)** Change in LH at 12 h (iU/L) was weakly negatively associated with change in progesterone at 12 h (nmol/L) after GnRHa by simple linear regression (n = 148). Change in progesterone at 12 h (nmol/L) = −0.202 × change in serum progesterone at 12 h + 60.98, r^2^ = 0.025, *P* = 0.05. **(F)** Change in LH at 12 h (iU/L) was negatively associated with change in progesterone at 12 h (nmol/L) after kisspeptin by simple linear regression (n = 110). Change in progesterone at 12 h (nmol/L) = −1.522 × change in serum progesterone at 12 h + 16.16, r^2^ = 0.036, *P* = 0.05.

Endogenous LH rise at 12 h following hCG was negatively associated with serum hCG (**Figure 4A**). Progesterone rise at 12 h was positively predicted by hCG level following hCG trigger (**Figure 4B**) but was negatively predicted by LH rise following all triggers (**Figures 4C–F**).

**Figure 5:**
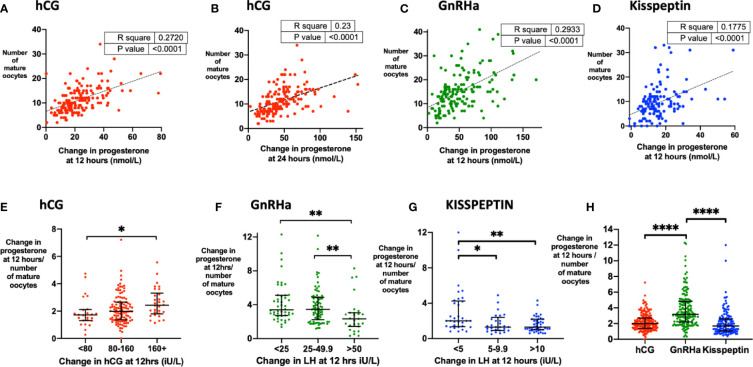
Progesterone rise during oocyte maturation. **(A)** Change in progesterone at 12 h (nmol/L) after hCG trigger predicted the number of mature oocytes retrieved by simple linear regression (n= 159). Number of mature oocytes retrieved = 0.204 x change in progesterone at 12 h (nmol/L) + 6.60, r^2^ = 0.27, *P <* 0.0001. **(B)** Change in progesterone at 24 h (nmol/L) after hCG trigger predicted the number of mature oocytes retrieved by simple linear regression (n= 161). Number of mature oocytes retrieved = 0.098 x change in progesterone at 24 h (nmol/L) + 6.8, r^2^ = 0.23, *P* < 0.0001. **(C)** Change in progesterone at 12 h (nmol/L) after GnRHa trigger predicted the number of mature oocytes retrieved by simple linear regression (n= 151). Number of mature oocytes retrieved = 0.133 x change in progesterone at 12 h (nmol/L) + 8.37, r^2^ = 0.29, *P* < 0.0001. One outlier has not been shown on the graph. **(D)** Change in progesterone at 12 h (nmol/L) after kisspeptin trigger predicted the number of mature oocytes retrieved by simple linear regression (n = 143). Number of mature oocytes retrieved = 0.297 x change in progesterone at 12 h (nmol/L) + 4.83, r^2^ = 0.18, *P <* 0.0001. **(E)** Median (IQR) of change in progesterone at 12 h (nmol/L) divided by number of mature oocytes after hCG by categories of change in serum hCG at 12 h (iU/L) (n = 159). Categories were compared by the Kruskal-Wallis test with post-hoc Dunn’s multiple comparison test. **(F)** Median (IQR) of change in progesterone at 12 h (nmol/L) divided by number of mature oocytes after GnRH by categories of change in LH at 12 h (iU/L) (n = 151). Categories were compared by the Kruskal-Wallis test with post-hoc Dunn’s multiple comparison test. One outlier has not been shown on the graph. **(G)** Median (IQR) of change in progesterone at 12 h (nmol/L) divided by number of mature oocytes after kisspeptin by categories of change in LH at 12 h (iU/L) (n = 151). Categories were compared by the Kruskal-Wallis test with post-hoc Dunn’s multiple comparison test. **(H)** Median (IQR) of change in progesterone at 12 h divided by number of mature oocytes after each trigger is presented: hCG (n = 159); GnRHa (n = 151), kisspeptin (n = 137). Categories were compared by Kruskal-Wallis test with *post hoc* Dunn’s multiple comparison test. Four outliers were not shown on the graph. *P < 0.05, **P < 0.01,****P < 0.0001.

The strongest biochemical predictor of the number of mature oocytes retrieved was the rise in serum progesterone. Progesterone rise at 12 h predicted the number of mature oocytes following hCG (r^2 =^ 0.23, P < 0.0001), GnRHa (r^2^ = 0.29, P < 0.0001) and kisspeptin (r^2^ 0.18, P < 0.0001) (**Figures 5A–D**). The relationship between MOY and progesterone rise at 12 h was much weaker (r^2^ hCG 0.07, GnRH 0.02, KP 0.02). To assess, whether each oocyte produced the same amount of progesterone during maturation after the different triggers we assessed progesterone rise per mature oocyte. Accordingly, progesterone rise at 12 h per mature oocyte was greater after GnRHa (3.18 nmol/L) than hCG (1.7 nmol/L) or kisspeptin (1.99 nmol/L) (**Figure 5H**). Similarly, the progesterone rise per mature oocyte was greater with hCG level following hCG trigger, but lesser with LH rise at 12 h following other triggers (**Figures 5E–G**).

Data comparing luteal estradiol and progesterone rises are presented in Supplemental Data.

## Discussion

We examined the hormonal responses following three triggers of oocyte maturation with distinct mechanisms of action to provide an insight into the endocrine requirements for oocyte maturation. HCG levels peaked at 24 h following hCG (albeit similar levels were encountered by 12 h), whereas LH-levels peaked sooner at 4 to 6 h following both GnRHa and kisspeptin. It is likely that a threshold level for LH-like activity needs to be reached to initiate the process of oocyte maturation. The timing of oocyte retrieval is precisely controlled to occur following oocyte maturation but prior to ovulation (2). Consequently, it is noteworthy that the same interval between trigger and oocyte retrieval is ordinarily used (36–37 h) following all three triggers despite the different times of peak LH-like activity. Although near maximal levels of hCG are achieved by 12 h, it is conceivable that the threshold to initiate oocyte maturation is exceeded even sooner after hCG administration, closer to the 4 h peak observed following GnRHa and kisspeptin to permit a similar duration for oocyte maturation to occur after each trigger.

We investigated the threshold for LH-like activity required for oocyte maturation. A lower level of LH-like activity was sufficient for efficacious oocyte maturation in some women, however oocyte maturation was more reliable with higher levels. Indeed, the proportion exceeding the median mature oocyte yield for each trigger was increased by the level of LH-like activity achieved. Although the LH surge following kisspeptin was of lower amplitude than GnRHa (15), it is possible that kisspeptin could enhance oocyte maturation *via* an additional direct action at ovarian kisspeptin receptors to compensate for the lower LH rise (25). Indeed, kisspeptin enhances *in vitro* maturation of ovine (26) and porcine (27) immature oocytes.

GnRHa and kisspeptin triggers must be used in the context of a GnRH antagonist co-treated stimulation protocol. Thus, the GnRH antagonist must be competitively displaced from the GnRH receptor in order to generate a gonadotropin rise. Consequently, a low LH level prior to the trigger could reflect increased suppression from the GnRH antagonist, and there are data to suggest that escape from GnRH antagonist suppression varies between individuals, occurring more precipitously with greater BMI (28). Thus, the attenuated LH rise following GnRHa and kisspeptin with lower pre-trigger LH values, could reflect more pronounced suppression by the GnRH antagonist. However, it is also recognized that the LH value prior to native GnRH determines subsequent LH rise even in the absence of GnRH antagonist pre-treatment (29). Indeed, the risk of encountering an LH <15 iU/L at 12 h following GnRHa (a threshold commonly used to denote a suboptimal LH rise), occurred in 17.3% of those with a pre-trigger LH <0.5 iU/L but only 3.8% if pre-trigger LH was >2 iU/L. Thus, adjusting the timing and dose of the GnRH antagonist to avoid excessive suppression could further optimize the efficacy of the trigger (30).

Similarly, the hCG level following hCG trigger was negatively associated with body weight consistent with published reports (26). The cohort of women who received hCG in this study was from Vietnam and had a lower body weight in comparison to western women. Thus, it is likely that western patients with higher body weights could encounter lower serum hCG levels more often, with a more detrimental impact on oocyte maturation than observed in the present study. Indeed, obese women have been reported to have an increased chance of encountering an hCG level <50 iU/L, with a subsequent increased risk of suboptimal oocyte maturation (31).

Interestingly, progesterone rose immediately following administration of the trigger and the level by 12 h was strongly predictive of the number of mature oocytes that would be retrieved. This was the case after all three triggers notwithstanding the fact that oocytes would not be mature had they been collected at this time-point. Logically, one would hypothesize that a greater LH rise following the trigger would result in increased oocyte maturation and a greater rise in progesterone. In fact, we observed that the LH rise after the trigger was inversely associated with progesterone rise. Progesterone is known to exert negative feedback on GnRH and LH during the luteal phase of the natural cycle (32, 33). As progesterone increased almost immediately following administration of the trigger, it is conceivable that progesterone tempered the LH rise following the trigger through negative feedback. Progesterone rise was strongly associated with the number of mature oocytes retrieved; therefore we examined the “progesterone rise per mature oocyte” and observed that this was greater for GnRHa than for either hCG or kisspeptin. This increased progesterone production during oocyte maturation may represent a specific characteristic of GnRHa triggering as the LH rise following GnRHa exceeds that by kisspeptin, which was associated with lower progesterone production.

Insufficient LH-like exposure increases the risk of “empty follicle syndrome”—a condition where no oocytes are retrieved (2). Early assessment of biochemical parameters to predict whether a mature oocyte is likely to be retrieved is therefore of value, as this can allow for re-administration of the trigger and rescheduling of oocyte retrieval to prevent a failure to retrieve oocytes. Logically, LH rise would be the measure that should confirm the successful deployment of the trigger. However, due to the inverse relationship of LH rise with progesterone rise and the closer relationship between progesterone rise and the number of mature oocytes retrieved, in fact progesterone rise appears to be the most reliable predictive biochemical marker of successful oocyte maturation.

Peak progesterone levels after hCG occurred at 3 days following oocyte retrieval, however 89% of women still exceeded the limit of detection for progesterone at 156 h (day 5 post oocyte retrieval) when a blastocyst transfer is most usually conducted, whereas progesterone levels after GnRHa were already low by this timepoint. The mid-luteal rise in progesterone after hCG far exceeded that generated during oocyte maturation by ~4-fold, whereas estradiol rose during the luteal phase to ~2.5-fold higher levels than trough levels at 60 h post administration. Nevertheless, this suggests that both estradiol and progesterone can be used to monitor corpora luteal function (34).

Strengths of the study include access to detailed endocrine data following each trigger. Limitations include the heterogenous nature of the study population. Further prospective study with direct comparison in the same population is indicated to verify the findings presented.

In summary, we evaluated the endocrine profile following each trigger and assess its impact on oocyte maturation. An unexpected negative association between LH and progesterone rises was observed during oocyte maturation. Moreover, progesterone rise appears to be the most reliable biochemical predictive marker for oocyte maturation following all triggers and the progesterone rise per oocyte was higher following GnRHa trigger compared to other agents. These findings further explicate our understanding of the endocrine changes during induction of oocyte maturation.

## Data Availability Statement

The datasets generated for this study are available on request to the corresponding author.

## Ethics Statement

The studies involving human participants were reviewed and approved by The Institutional Review Board (IRB) at My Duc Hospital, Ho Chi Minh City, Vietnam and Hammersmith and Queen Charlotte’s Research Ethics Committee, London. The patients/participants provided their written informed consent to participate in this study.

## Author Contributions

AA, TH, PH, LV, and WD designed the study. AA, TH, VH, SC, AC, CI-E, TH, GT, RS, PH, LV, and WD contributed to collection of the data. AA, TH, AH, and TK analyzed the data. All authors were responsible for the reporting of results and writing of the manuscript. LV and WD provided overall oversight. All authors contributed to the article and approved the submitted version.

## Funding

The study was designed, conducted, analyzed and reported entirely by the authors. Data from clinical trials using kisspeptin was funded by independent research grants from the MRC, and NIHR and supported by the NIHR/Wellcome Trust Imperial Clinical Research Facility and Imperial Biomedical Research Centre. The views expressed are those of the author(s) and not necessarily those of the MRC, the NHS, the NIHR or the Department of Health. The Section of Endocrinology and Investigative Medicine is funded by grants from the MRC, BBSRC, NIHR, an Integrative Mammalian Biology (IMB) Capacity Building Award, an FP7-HEALTH-2009-241592 EuroCHIP grant and is supported by the NIHR Biomedical Research Centre Funding Scheme. Data from trials of GnRH agonist/hCG data were funded by My Duc Hospital, Ho Chi Minh City, Vietnam. AA is funded by an NIHR Clinician Scientist Award (CS-2018-18-ST2-002). CI-E is funded by an MRC Clinical Research Training Fellowship (MR/M004171/1). TK is supported by EPSRC EP/P015638/1. WD is funded by an NIHR Professorship (CDF-2009-02-05) and MRC grant (G1000455).

## Conflict of Interest

AA and WD have undertaken consultancy work for Myovant Sciences Ltd who are developing a kisspeptin receptor agonist.

The remaining authors declare that the research was conducted in the absence of any commercial or financial relationships that could be construed as a potential conflict of interest.

The reviewer AC declared a past co-authorship with one of the authors PH to the handling editor
