# Supplementary material for: Endocrine Requirements for Oocyte Maturation Following hCG, GnRH Agonist, and Kisspeptin During IVF Treatment
Source: Front Endocrinol (Lausanne). 2020 Oct 6;11:537205. doi: 10.3389/fendo.2020.537205 (PMC7573298; doi:10.3389/fendo.2020.537205)
Supplement: Supplementary file 1 [file DataSheet_1.pdf]

Supplemental Data

Luteal estradiol and progesterone-rise after GnRHa and hCG

Estradiol levels peaked during the first 12 h after hCG followed by a trough at 60 h (2.5 days) after administration, prior to a secondary 2.5-fold increase at 132 h (5.5 days) (**Figure 1C**). Progesterone levels following hCG were positively associated with estradiol levels at 12, 24, 36, 60, 84, 156, and 180 h ($P<0.015$ for all) (**Supplemental Figure 3A & 6B**). The ratios of estradiol to progesterone level were 293:1, 86:1, 51:1, 8:1, 9:1, 18:1, 16:1 at 12, 24, 36, 60, 84, 156, 180 h, respectively. Furthermore, progesterone and estradiol levels at later time-points correlated with levels of the same hormone at earlier time-points, and with the number of follicles of 12-19 mm at the time of trigger (**Supplemental Figure 3C**). This supports the assertion that mid-luteal progesterone levels are derived from the same cohort of follicles that give rise to early progesterone-rise during oocyte maturation, and subsequently form corpora lutea after hCG (**Supplemental Figure 3B & 3C**) or GnRHa (**Supplemental Figure 3D**).

The upper limit of detection for progesterone (>190 nmol/L) was exceeded during the mid-luteal phase following hCG, by 27% at 60 h, 76% at 84 h, 96% at 108 h, 95% at 132 h, 89% at 156 h, and 61% at 180 h. Consequently, linear regression to peak progesterone values during the mid-luteal phase following hCG were less instructive. Progesterone-rises at 132 h and at 180 h following hCG were positively associated with serum hCG at 36, 60, 84, 108 and 132 h ($P<0.03$ for all), but not at 12 or 24 h.

By comparison, the peak secondary progesterone-rise following GnRHa occurred earlier at 84 h to a median (IQR) of 55.0 (32.8, 82.1 nmol/L), with only 4% exceeding 190 nmol/L. This peak progesterone level at 84 h did not correlate with LH-rise at earlier timepoints following GnRHa ($P>0.36$ for all). Progesterone-rises at 132 h or 180 h following GnRHa were positively associated with LH-rises at 12, 24 and 36 h ($P<0.007$ for all), but not at 84 h or 132 h.

We assessed whether hCG/LH levels were associated with maintenance of progesterone levels from peak levels during oocyte maturation to the secondary rise at the mid-luteal phase. Women with a progesterone level >150 nmol/L at 180 h following hCG (or a rise of >100 nmol/L from the peak progesterone at 24 h) had higher hCG levels at 60 h onwards (but not at earlier timepoints) (**Supplemental Figure 3E**). A fall in progesterone of >10 nmol/L from 24 h to 84 h was associated with lower LH levels at 4 h after GnRHa (LH 127 iU/L) than remaining patients (LH 153 iU/L) *($P=0.009$)*. Furthermore, women with higher LH values at 84 h following GnRHa maintained more similar progesterone values during the luteal phase as at 24 h (**Supplemental Figure 3F**). Similarly, the fall in progesterone between 24 h and 132 h was positively predicted by LH at 36 h, 84 h and 132 h ($P<0.0005$ for all). Accordingly, to maintain similar progesterone levels at 132 h as at 24 h, was associated with an LH of 5 iU/L at 36 h and 1.2-1.5 iU/L at 84-132 h.

Supplementary Figure Legends

Supplementary Figure 1- Predicting mature oocytes.

(A) Serum hCG at 24 h (iU/L) after hCG trigger was not associated with the number of mature oocytes retrieved by simple linear regression (n=161). Number of mature oocytes retrieved = 0.004 x serum hCG at 24 h (iU/L) + 10.81, r^2=0.001, *P=0.63*. (B) Change in LH at 4 h (iU/L) after GnRHa was not associated with the number of mature oocytes retrieved by simple linear regression (n=150). Number of mature oocytes retrieved = 0.009 x change in LH at 4 h (iU/L) + 14.53, r^2=0.002, *P=0.59*. (C) Change in LH at 12 h (iU/L) after GnRHa was not associated with the number of mature oocytes retrieved by simple linear regression (n=151). Number of mature oocytes retrieved = 0.02 x change in LH at 12 h (iU/L) + 14.58, r^2=0.006, *P=0.34*. (D) Change in LH at 12 h (iU/L) after kisspeptin was weakly associated with the number of mature oocytes retrieved by simple linear regression (n=143). Number of mature oocytes retrieved = 0.18 x change in LH at 12 h (iU/L) + 8.2, r^2=0.03, *P=0.048*. (E) Serum hCG at 12 h (iU/L) versus the cumulative mature oocyte yield (%) following hCG (n=161). (F) Serum hCG at 24 h (iU/L) versus the cumulative mature oocyte yield (%) following hCG (n=161). (G) Change in LH at 12 h (iU/L) versus the cumulative mature oocyte yield (%) following GnRH (n=151). (H) Change in LH at 12 h (iU/L) versus the cumulative mature oocyte yield (%) following Kisspeptin (n=143).

Supplementary Figure 2- Oocyte maturation rate and fertilization rate.

(A) Median (IQR) of the oocyte maturation rate (%) by serum hCG at 24 h (iU/L) following hCG (n=161). Categories were compared by Kruskal-Wallis test with post-hoc Dunn's multiple comparison test. (B) Median (IQR) of the oocyte maturation rate (%) by serum LH at 12 h (iU/L) following GnRHa (n=124). Categories were compared by Kruskal-Wallis test

with post-hoc Dunn's multiple comparison test. (C) Median (IQR) of the oocyte maturation rate (%) by serum LH at 12 h (iU/L) following kisspeptin (n=139). Categories were compared by the Kruskal-Wallis test with post-hoc Dunn's multiple comparison test. (D) Median (IQR) of the fertilization rate (%) by serum hCG at 24 h (iU/L) following hCG (n=161). Categories were compared by the Kruskal-Wallis test with post-hoc Dunn's multiple comparison test. (E) Median (IQR) of the oocyte maturation rate (%) by serum LH at 12 h (iU/L) following GnRHa (n=152). Categories were compared by the Kruskal-Wallis test with post-hoc Dunn's multiple comparison test. (F) Median (IQR) of the oocyte maturation rate (%) by serum LH at 12 h (iU/L) following kisspeptin (n=142). Categories were compared by the Kruskal-Wallis test with post-hoc Dunn's multiple comparison test.

Supplemental Figure 3- Progesterone levels during the luteal phase.

(A) Serum progesterone at 24 h (nmol/L) was positively predicted by serum estradiol at 24 h (pmol/L) after hCG (n=161). Serum progesterone at 24 h = 0.003 x serum estradiol at 24 h + 38.14, r^2=0.14, *P<0.0001.* (B) Serum progesterone at 60 h (nmol/L) was positively predicted by serum estradiol at 60 h (pmol/L) after hCG (n=161). Serum progesterone at 60 h = 0.025 x serum estradiol at 60 h + 113.9, r^2=0.1706, *P<0.0001.* (C) Number of follicles 12-19mm was positively predicted by change in progesterone at 60 h (pmol/L) after hCG (n=161). Number of follicles 12-19mm = 0.066 *X + 3.050, r^2=0.4475, *P<0.0001.* (D) Serum progesterone at 84 h (nmol/L) was positively predicted by serum estradiol at 84 h (pmol/L) after GnRH (n=118). Serum progesterone at 84 h = 0.025 x serum estradiol at 60 h + 40.29. r^2=0.159, *P<0.0001.* (E) Change in hCG (iU/L) over time by categories of progesterone at 180 h (< 150 nmol/L / >150 nmol/L) after hCG. A mixed effects analysis was performed. (F) Change in progesterone from 24-132 h (nmol/L) was positively associated with serum LH at 84 h (iU/L). Categories were

compared by the Kruskal-Wallis test with post-hoc Dunn's multiple comparison test. $^{**}P<0.01$, $^{***}P<0.001$, $^{****}P<0.0001$.

Supplementary Table 1-

	Trigger			
	hCG (N=161)	GnRHa (N=165)	Kisspeptin (N=173)	P Value
**Age (yrs)**	32.3 ± 3.2	27.0 ± 4.3	30.5 ± 2.8	*P<0.001*
**Ethnicity**	Southeast Asian 100%	Southeast Asian 100%	Caucasian 62.6%<br>South Asian 28.2%<br>Afro-caribbean 4.6%<br>Other 4.6%	*P<0.001*
**Mass (kg)**	50.3 ± 5.7	51.3 ± 7.4	64.4 ± 9.4	*P<0.001*
**BMI (kg/m^2)**	20.5 ± 2.1	20.8 ± 2.7	24.2 ± 3.2	*P<0.001*
**Serum AMH (ng/ml)**	4.2 (26, 5.9)	6.4 (4.6, 9.4)	6.1 (3.5, 9.5)	*P<0.001*
**Antral Follicle Count**	7 (5, 10)	17 (13, 24)	31 (25, 44)	*P<0.001*
**Number of follicles on day of trigger**	14 (11, 16)	17 (13, 24)	27 (21, 39)	*P<0.001*
**Cumulative dose of recombinant FSH (IU)***	2400 (2025, 2700)	900 (700, 1300)	1750 (1388, 4225)	*P<0.001*

Supplementary Table 1 Baseline characteristics: Parametric variables are presented as mean ± standard deviation whilst non-parametric variables are presented as median (interquartile range). Parametric variables were compared by one-way ANOVA with *post hoc* Tukey's and non-parametric variables by Kruskal Wallis test with *post hoc* Dunn's test. Significant *P-values* on *post hoc* analysis were as follows: Age- hCG vs GnRH *P<0.0001*, hCG vs kisspeptin (KP) *P=0.001*, GnRHa vs KP *P<0.0001*. Body mass- KP vs GnRHa *P<0.0001*, KP vs hCG *P<0.0001.* Body mass index *(*BMI)- hCG vs KP *P<0.0001,* GnRHa vs KP *P<0.0001.* Anti-Müllerian hormone (AMH)- hCG vs GnRHa *P<0.0001,* hCG vs KP *P<0.0001.* Antral Follicle count- KP vs hCG *P<0.0001,* hCG vs GnRHa *P<0.0001*. Number of follicles on day of trigger KP vs GnRHa *P<0.0001*, KP vs hCG *P<0.0001,* hCG vs GnRHa *P=0.0009*. Cumulative dose of recombinant FSH KP vs GnRHa *P<0.0001*, KP vs hCG *P<0.0001,* hCG vs GnRHa *P<0.0001.*
