# Supplementary figures and images for: Endocrine Requirements for Oocyte Maturation Following hCG, GnRH Agonist, and Kisspeptin During IVF Treatment
Source: Front Endocrinol (Lausanne). 2020 Oct 6;11:537205. doi: 10.3389/fendo.2020.537205 (PMC7573298; doi:10.3389/fendo.2020.537205)

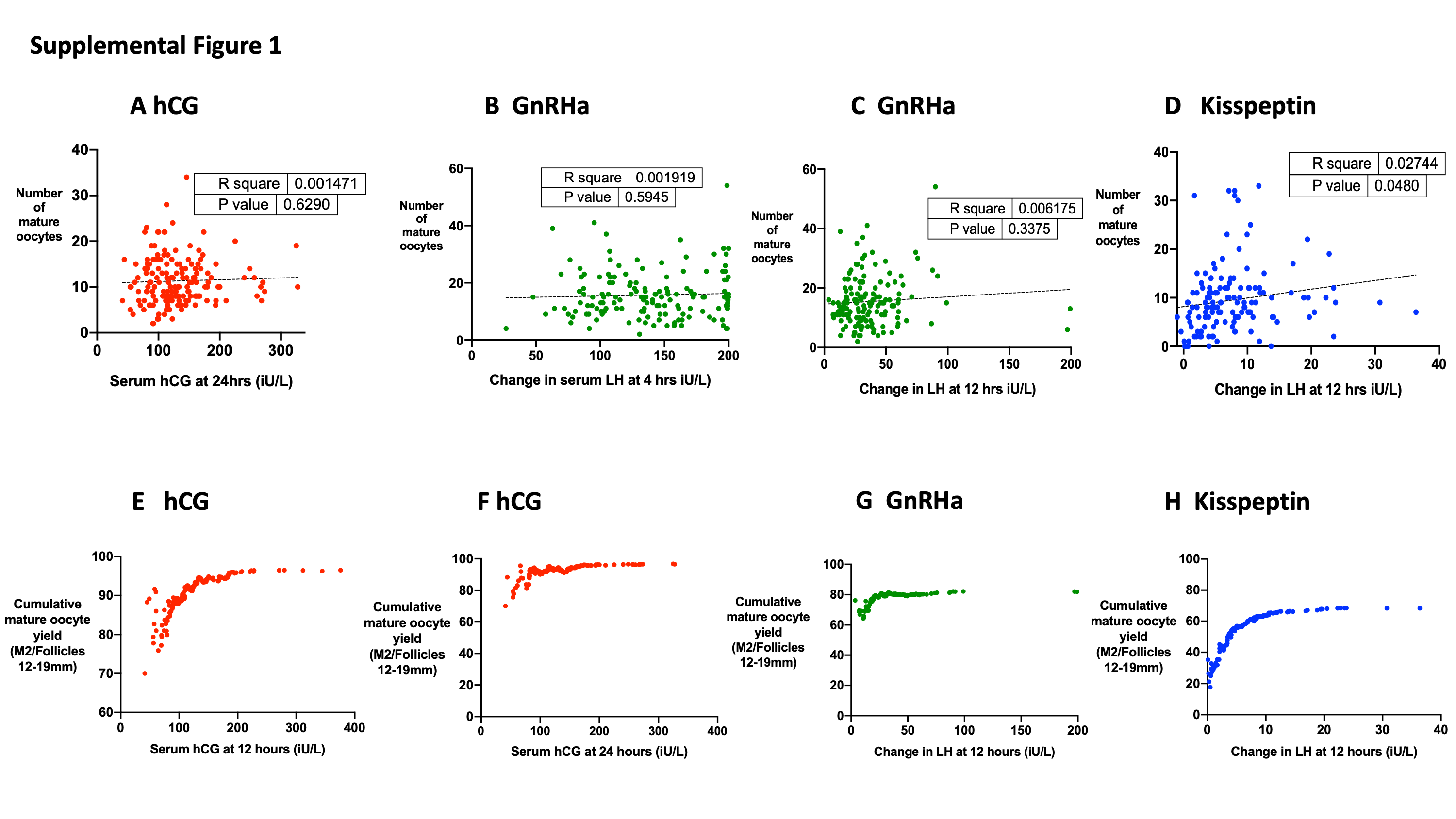

Supplement: Supplementary file 2 [file Image_1.tiff]

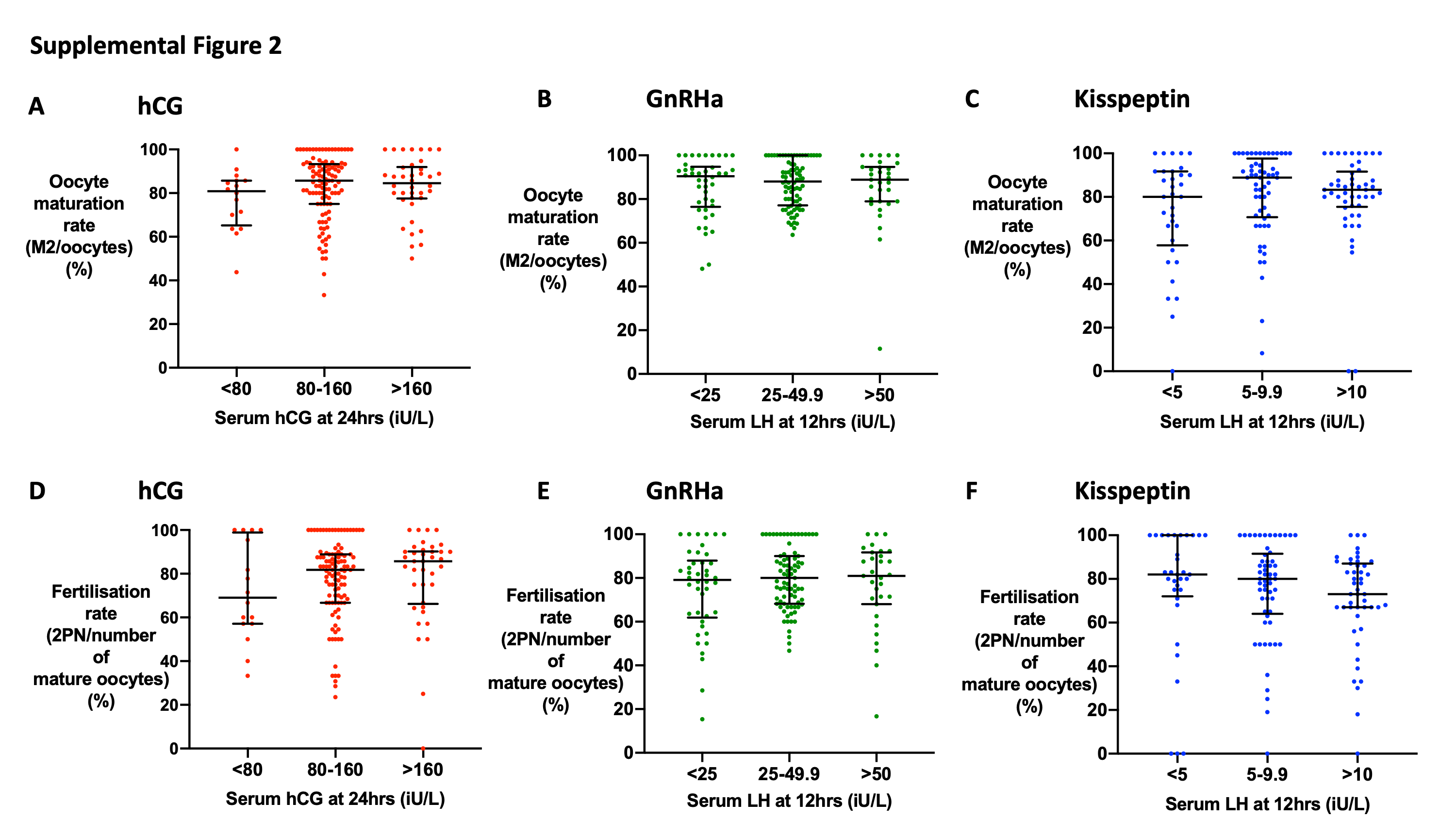

Supplement: Supplementary file 3 [file Image_2.tiff]

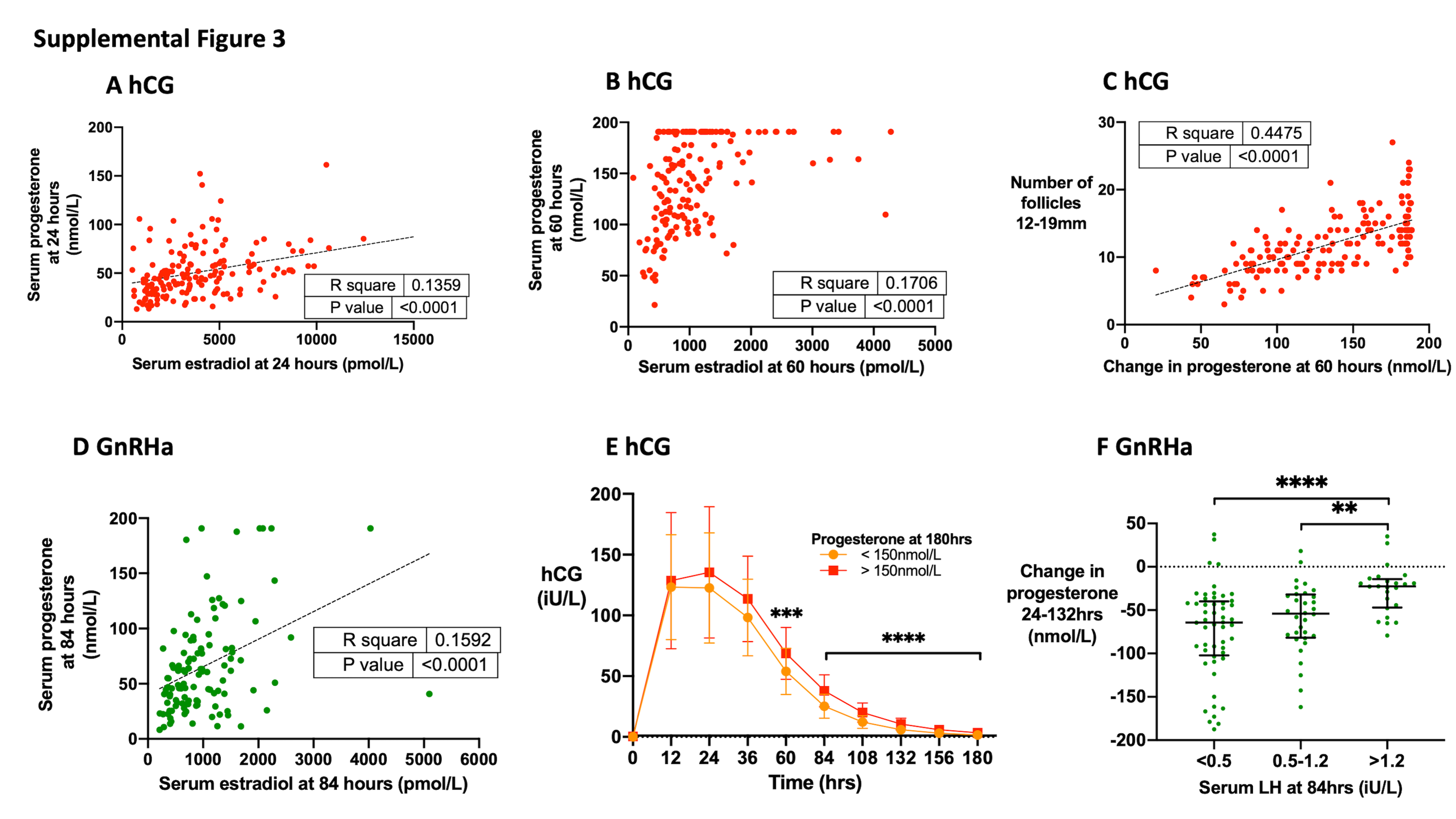

Supplement: Supplementary file 4 [file Image_3.tiff]
